# Cancer-associated oxidoreductase ERO1-α promotes immune escape through up-regulation of PD-L1 in human breast cancer

**DOI:** 10.18632/oncotarget.14960

**Published:** 2017-02-01

**Authors:** Tsutomu Tanaka, Goro Kutomi, Toshimitsu Kajiwara, Kazuharu Kukita, Vitaly Kochin, Takayuki Kanaseki, Tomohide Tsukahara, Yoshihiko Hirohashi, Toshihiko Torigoe, Yoshiharu Okamoto, Koichi Hirata, Noriyuki Sato, Yasuaki Tamura

**Affiliations:** ^1^ Department of Pathology, Sapporo Medical University, Sapporo, Japan; ^2^ Department of Clinical Veterinary Medicine, The United Graduate School of Veterinary Sciences, Yamaguchi University, Yamaguchi, Japan; ^3^ Department of Surgery, Sapporo Medical University, Sapporo, Japan; ^4^ Joint Department of Veterinary Medicine, Tottori University, Tottori, Japan; ^5^ Department of Molecular Therapeutics, Institute for the Business-Regional Collaboration, Center for Food & Medical Innovation, Hokkaido University, Sapporo, Japan

**Keywords:** ERO1-α, PD-L1, disulfide bond, triple negative breast cancer, oxidoreductase

## Abstract

Many human cancers have been reported to have enhanced expression of the immune checkpoint molecule programmed death-ligand 1 (PD-L1), which binds to programmed cell death-1 (PD-1) expressed on immune cells. PD-L1/PD-1 plays a role in inhibition of antitumor immunity by inducing T cell apoptosis and tolerance. Thus, it is crucial to elucidate mechanisms of PD-L1 expression on cancer cells. ERO1-α is an oxidase located in the endoplasmic reticulum. It is overexpressed in a variety of tumor types and it plays a role in disulfide bond formation in collaboration with PDI. Here, we investigated the influence of ERO1-α on expression of PD-L1 and immune escape. We demonstrated that ERO1-α augmented the expression of PD-L1 via facilitation of oxidative protein folding within PD-L1. In addition, we showed that overexpression of ERO1-α increased HIF-1α protein expression, resulting in an increase of PD-L1 mRNA as well as protein. In clinical cases, we observed that the expression of ERO1-α in triple negative breast cancer was related to the expression of PD-L1. Moreover, apoptosis of Jurkat leukemia T cells, which express PD-1, induced by tumor PD-L1 was inhibited when ERO1-α was depleted. The results suggest that targeting ERO1-α in tumor cells can be a novel approach for cancer immunotherapy. Therefore, the role of ERO1-α in tumor-mediated immunosuppression should be further explored.

## INTRODUCTION

Breast cancer has been the most common cancer worldwide in women over the past decade [1]. Triple negative breast cancer (TNBC), which lacks expression of the estrogen receptor, progesterone receptor and HER2/neu (HER2), accounts for 10% to 20% of breast cancer cases [2, 3]. TNBC is generally characterized by a high rate of distant metastasis and poorer disease-specific survival than that of other breast cancer subtypes [4].

An immunosuppressive condition, particularly in a tumor-associated microenvironment, is one of major obstacles for the development of effective immunotherapy. Tumors have developed strategies to evade antitumor immunity, and the developing tumor often co-opts the host immune system for its own tumor-promoting purpose. Much attention has recently been paid to immune checkpoint molecules including programmed death-ligand 1 (PD-L1) as immunosuppressive molecules. Expression of PD-L1 is found in various types of cells and tissues including the placenta, vascular endothelium, pancreatic islet cells, muscle, hepatocytes, and mesenchymal stem cells [5]. PD-L1 mRNA expression is elevated by interferon β [6], γ [7] and under a condition of hypoxia [8, 9]. PD-L1 binds to programmed cell death-1 (PD-1), a CD28 family receptor that is expressed on activated T cells, activated B cells and myeloid cells [10], and PD-L1/PD-1 plays a role in inhibition of autoimmunity by inducing T cell apoptosis [11] and inducing tolerance [12]. Enhanced expression of PD-L1 has been found in human cancers including glioblastoma, melanoma, lung cancer, ovary cancer, colon cancer and breast cancer [10]. Importantly, the expression of PD-L1 in TNBC is enhanced compared with that in other types of breast cancer [4, 13]. Some clinical studies using an anti-PD-L1 mAb have recently been performed [14, 15]. Brahmer et al. reported that blocking PD-L1 by using an anti-PD-L1 mAb results in both durable tumor regression (objective response rate, 6% to 17%) and prolonged (≧24 weeks) disease stabilization in patients [15]. Therefore, it is important to clarify underlying mechanisms for PD-L1 expression and develop a strategy for the regulation of PD-L1 expression in cancer immunotherapy.

Endoplasmic reticulum oxidoreductase 1-α (ERO1-α) is an oxidoreductase that exists in the endoplasmic reticulum (ER). ERO1-α plays a central role in disulfide bond formation of secreted and cell surface molecules [16–20] in collaboration with protein disulfide isomerase (PDI). A disulfide bond is produced at the post-translational level and is required for proper conformation and function of these molecules [20, 21]. In fact, we have recently demonstrated that expression of G-CSF, CXCL1, CXCL2 and MHC class I is regulated by proper oxidative protein folding by ERO1-α [22, 23]. We found that various types of tumors expressed high levels of ERO1-α and that the expression of ERO1-α in breast cancer is a poor prognosis factor [22–24]. However, the mechanisms by which ERO1-α expression affects the prognosis of breast cancer are still unclear. In this study, we found that ERO1-α up-regulates PD-L1 expression via not only oxidative protein folding but also unexpected up-regulation of PD-L1 mRNA expression through augmentation of hypoxia-inducible factor-1α (HIF-1α) protein expression in human TNBC cell lines. Knockdown of ERO1-α resulted in a significant attenuation of PD-L1-mediated T cell apoptosis, thus providing a lead for therapeutic modulation of hypoxia-mediated immunoresistance.

## RESULTS

### ERO1-α upregulates the expression of PD-L1 via both oxidative protein folding and increasing the mRNA of PD-L1

**Figure 1:**
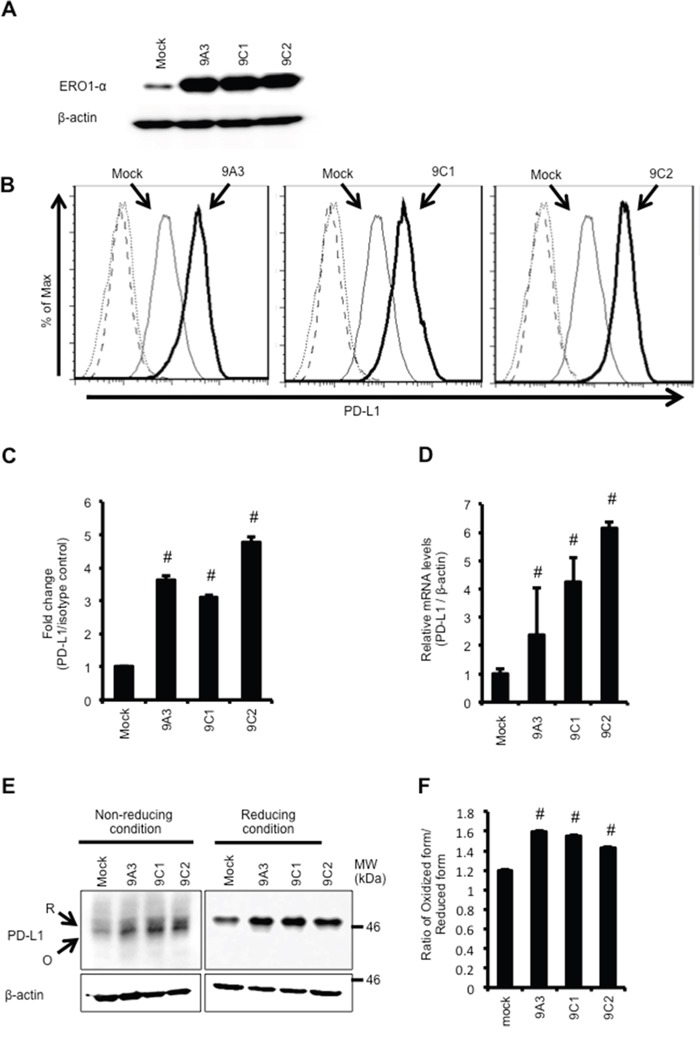
Overexpression of ERO1-α upregulates PD-L1 expression via accumulation of HIF-1α **A**. Western blot analysis of MDA-MB-231 mock and ERO1-α-overexpressed (OE: 9A3, 9C1 and 9C2) cells. **B**. Flow cytometric analysis of PD-L1 expression on mock (thin line) and OE cells (left: 9A3, middle: 9C1, right: 9C2; bold line). These cells incubated with a PE-labeled isotype control served as background controls (dotted line and dashed line). **C**. The mean fluorescence intensity (MFI) value obtained in mock cells was set to 1, and differences in MFI caused by 9A3 cells, 9C1 cells and 9C2 cells were plotted. **D**. PD-L1 mRNA levels in mock and OE cells determined by real-time PCR. **E**. Redox status of PD-L1 was examined by Western blotting under reducing or non-reducing conditions. Reduced form (R) or oxidized form (O) of PD-L1 is indicated. **F**. The ratio of the oxidized form and reduced form of PD-L1 was obtained by densitometry analysis. Data are shown from single experiments representative of three experiments performed. ^#^p<0.05, Dunnett's test.

We have shown that the expression of ERO1-α in human breast cancer tissue and in cell lines was augmented compared with that in normal breast tissue [22, 25]. In this study, we focused on how ERO1-α affects expression of the immune checkpoint molecule PD-L1 in TNBC because patients with TNBC generally have a poor prognosis. Therefore, we examined the role of ERO1-α in the expression of PD-L1 using TNBC line MDA-MB-231 cells. We established ERO1-α-overexpressed (OE: 9A3, 9C1 and 9C2) cells by introducing human cDNA of ERO1-α (Figure 1A). When we compared mock cells with OE cells, we found by flow cytometry that the surface expression of PD-L1 in all OE cell lines was augmented (Figure 1B, 1C). These results suggested that the expression of ERO1-α upregulated PD-L1 expression. We then compared PD-L1 mRNA expression levels in mock and OE cells by real-time RT-PCR. Unexpectedly, we found that PD-L1 mRNA expression levels in OE cells were significantly higher than those in mock cells (Figure 1D). Since recent studies have demonstrated that PD-L1 mRNA expression level is regulated by HIF-1α [8, 9], we examined the expression levels of HIF-1α. We found that the protein levels of HIF-1α in OE cells were higher than those in mock cells (Supplementary Figure 1A), indicating that HIF-1α may play a role in the upregulation of PD-L1 mRNA expression. To further examine the role of HIF-1α in OE cells, we silenced HIF-1α in 9C2 (9C2 siHIF-1α) cells using siRNA for HIF-1α (Supplementary Figure 1B). When we compared the PD-L1 mRNA expression level in 9C2 cells with the levels in control siRNA cells (9C2 siNC cells) and 9C2 siHIF-1α cells, we observed that the PD-L1 mRNA expression level in 9C2 siHIF-1α cells was decreased compared with that in 9C2 siNC cells (Supplementary Figure 1C). These results indicated that ERO1-α-mediated upregulation of HIF-1α augmented the mRNA expression of PD-L1. It has been shown that intracellular reactive oxygen species (ROS) increase HIF-1α protein levels by inhibiting HIF-prolyl hydroxylase (PHD) activity [26, 27]. In addition, ERO1-α has been shown to produce ROS during oxidation of a substrate protein [28]. Therefore, we compared the levels of ROS in mock cells and OE cells. Fluorescence microscopic analysis and flow cytometric analysis using CM-H_2_DCFDA showed that overexpression of ERO1-α in OE cells increased the levels of ROS compared with those in mock cells (Supplementary Figure 1D, 1E). Moreover, we found that OE cells had decreased levels of hydroxyl-HIF-1α, indicating that HIF-1α escaped from degradation by proteasome (Supplementary Figure 1F). These results suggested that expression of ERO1-α results in accumulation of HIF-1α protein via production of ROS, leading to the increased expression of PD-L1 mRNA. Next, we compared the protein levels of PD-L1 by Western blot analysis. Since ERO1-α acts as an oxidoreductase and PD-L1 protein has an intramolecular disulfide bond [29], we investigated the redox status of PD-L1 in mock cells and OE cells by Western blot analysis under a non-reducing condition using N-ethylmaleimide (NEM). As expected, we found that the ratio of the oxidized form (mature form) to the reduced form (immature form) of PD-L1 in OE cells was significantly higher than that in mock cells (Figure 1E, 1F). These results suggested that ERO1-α up-regulates the expression of PD-L1 via not only oxidative protein folding but also promoting HIF-1α-mediated mRNA expression.

### Knockdown of ERO1-α downregulates PD-L1 expression via decreased oxidative protein folding

**Figure 2:**
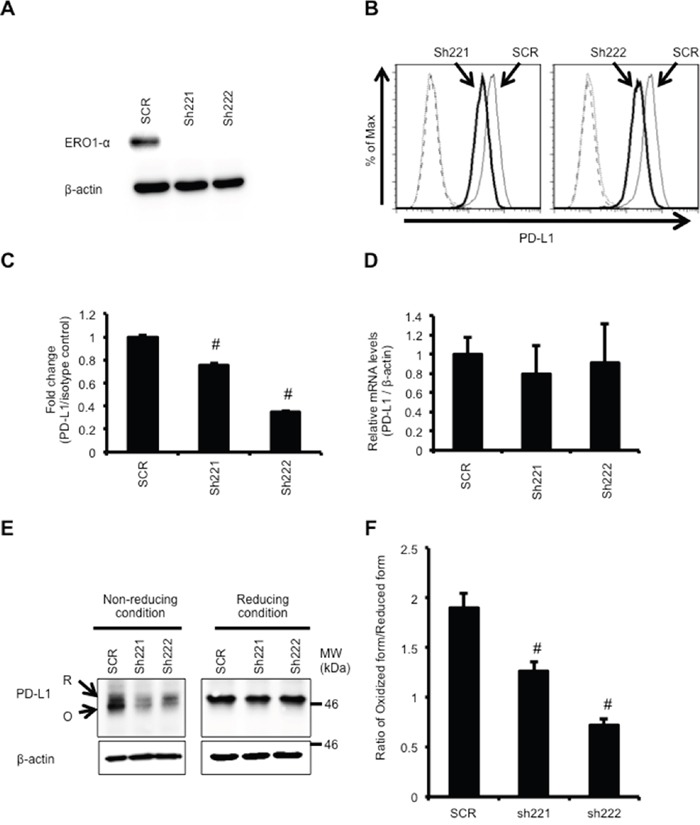
Knockdown of ERO1-α downregulates PD-L1 expression via decreased oxidative protein folding **A**. Western blot analysis of MDA-MB-231 scrambled shRNA-transfected (SCR) and ERO1-α knockdown (KD: sh221 and sh222) cells. **B**. Flow cytometric analysis of PD-L1 expression on SCR (thin line) and KD cells (left: sh221, right: sh222; bold line). These cells incubated with a PE-labeled isotype control served as background controls (dotted line and dashed line). **C**. The mean fluorescence intensity (MFI) value obtained in SCR cells was set to 1, and differences in MFI caused by sh221 cells and sh222 cells were plotted. **D**. PD-L1 mRNA levels in SCR and KD cells determined by real-time PCR. **E**. Redox status of PD-L1 was examined by Western blotting under reducing or non-reducing conditions. Reduced form (R) or oxidized form (O) of PD-L1 is indicated. **F**. The ratio of the oxidized form and reduced form of PD-L1 was obtained by densitometry analysis. Data are shown from single experiments representative of three experiments performed. ^#^p<0.05, Dunnett's test.

To further confirm the effect of ERO1-α on expression of PD-L1, we generated MDA-MB-231 cells with ERO1-α knockdown (KD: sh221 and sh222) using shRNA against ERO1-α (Figure 2A). Scrambled control (SCR) cells were transfected with scrambled control shRNA. When we compared SCR cells and KD cells, we found by flow cytometry that the surface expression of PD-L1 in KD cells was significantly decreased (Figure 2B, 2C). Next, we compared PD-L1 mRNA expression levels by real-time RT-PCR. The mRNA expression levels were not different in SCR cells and KD cells (Figure 2D). We then compared the protein levels of PD-L1 by Western blot analysis. We found that the ratio of the oxidized form (mature form) to the reduced form (immature form) of PD-L1 in KD cells was significantly lower than that in SCR cells under a non-reducing condition (Figure 2E, 2F). Thus, knockdown of ERO1-α resulted in decreased expression of PD-L1 through insufficient oxidative protein folding. However, since there was a discrepancy in the context of mRNA expression of PD-L1 between overexpression and knockdown of ERO1-α, we compared the levels of ROS (Supplementary Figure 2A) as well as HIF-1α protein levels (Supplementary Figure 2B) in SCR cells and KD cells. We found that both levels of ROS and HIF-1α protein levels were decreased in KD cells compared with the levels in SCR cells. We also examined whether HIF-1α regulates PD-L1 mRNA expression levels in in a steady state of MDA-MB-231 WT cells, because MDA-MB-231 WT cells expressed moderate levels of ROS and HIF-1α protein as shown in Supplementary Figure 2A, 2B. Unexpectedly, knockdown of HIF-1α in WT cells using siRNA to HIF-1α did not affect the PD-L1 mRNA level (Supplementary Figure 2C, 2D). These results suggest that PD-L1 mRNA in a steady state of WT cells, SCR cells and KD cells is regulated by factors other than HIF-1α. In contrast, in MDA-MB-231 OE cells, PD-L1 mRNA expression levels were regulated by factors including HIF-1α. In fact, it has been shown that the PD-L1 mRNA expression level is regulated by NF-κB [30], interferon regulatory factor (IRF)-1 [31], STATs [32, 33], MEK/ERK [32, 34] and AP-1 [30].

To generalize these findings, we examined the effect of ERO1-α on the expression of PD-L1 using another human TNBC cell line, MDA-MB-468. Knockdown of ERO1-α resulted in decreased expression of PD-L1 in MDA-MB-468 cells compared with that in MDA-MB-468 cells treated with control siRNA. (Supplementary Figure 3A-3C). In addition, PD-L1 mRNA expression levels were the same in control cells and ERO1-α knockdown cells when we used MDA-MB-468 cells (Supplementary Figure 3D).

### ERO1-α plays a critical role in IFN-γ-induced upregulation of PD-L1 expression in tumor cells

A previous study demonstrated that PD-L1 expression in cancer cells is enhanced by IFN-γ [35]. To investigate the role of ERO1-α in the expression of PD-L1 in cells treated with IFN-γ, we treated SCR cells and sh221 cells with IFN-γ (100 ng/ml) for 24 h and then compared the cell surface expression of PD-L1 in these cells using flow cytometry. We found that IFN-γ treatment increased the cell surface expression of PD-L1 in SCR cells compared with that in cells without IFN-γ treatment (Supplementary Figure 4A, 4B). In contrast, the expression of PD-L1 in sh221 cells treated with IFN-γ was not altered when compared with sh221 cells without IFN-γ treatment. We then compared the PD-L1 mRNA expression levels in these cells by using real-time PCR. We found that IFN-γ treatment significantly and equally upregulated PD-L1 mRNA expression in SCR cells and sh221 cells (Supplementary Figure 4C, 4D). These results suggested that ERO1-α also plays a critical role in the IFN-γ-induced upregulation of PD-L1 expression.

### Functional inhibition of ERO1-α by using the ERO1-α inhibitor EN460 leads to decreased expression of PD-L1 via inhibition of oxidative protein folding

**Figure 3:**
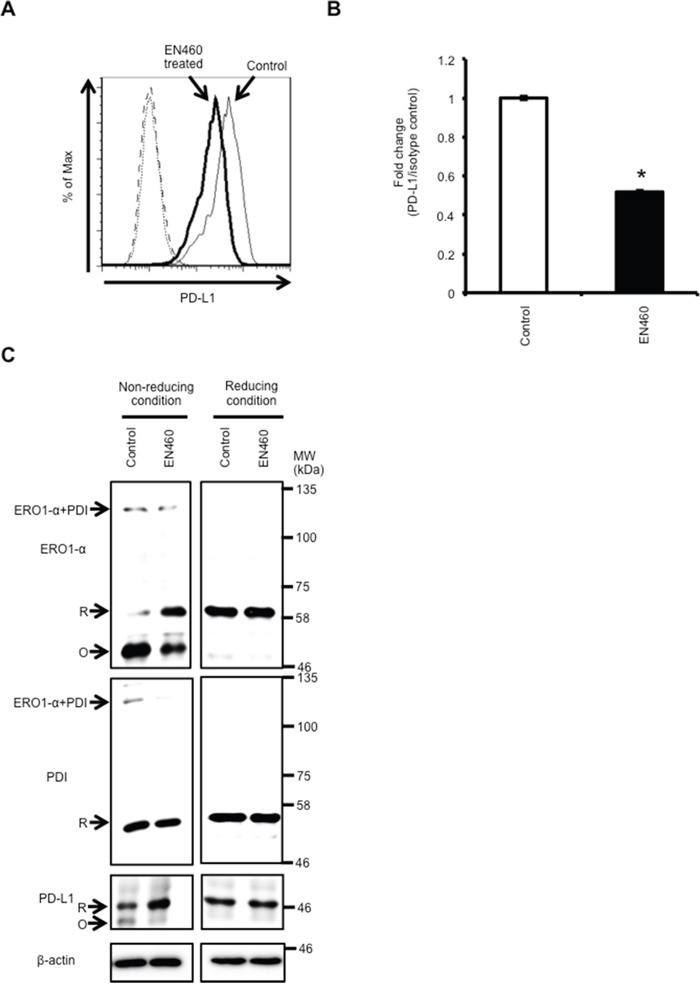
Functional inhibition of ERO1-α by using the ERO1-α inhibitor EN460 caused a decrease in the expression of PD-L1 **A**. Flow cytometric analysis of PD-L1 expression on control cells (thin line) and MDA-MB-231 cells treated with the ERO1-α inhibitor EN460 (12.5μM) (bold line). These cells incubated with a PE-labeled isotype control served as background controls (dotted line and dashed line). **B**. The ratios of MFI of PD-L1 and the isotype control were compared in control cells and EN460 (12.5 μM) -treated cells. **C**. Redox status of ERO1-α, PDI, and PD-L1 was examined by Western blotting under reducing or non-reducing conditions. Reduced form (R) or oxidized form (O) of each protein is indicated. * *p*<0.001, unpaired Student's t-test. Data are shown from single experiments representative of four experiments performed.

Since the ERO1-α inhibitor EN460 is known to inhibit oxidative protein folding by reductive inactivation of ERO1-α [36], we treated MDA-MB-231 WT cells with EN460 to further examine the effect of ERO1-α on expression of PD-L1. We found by flow cytometric analysis that the surface expression of PD-L1 in MDA-MB-231 WT cells treated with EN460 was decreased compared with that in control cells (Figure 3A, 3B). However, PD-L1 mRNA expression levels were not different in control cells and treated cells (data not shown). The results indicated that functional inhibition of ERO1-α did not influence the PD-L1 gene expression level. Next, we compared the protein levels by Western blot analysis under a non-reducing condition using methyl methanethiosulfonate (MMTS). We found that the ratio of the oxidized form (mature form) to the reduced form (immature form) of PD-L1 in treated cells was significantly decreased compared with that in control cells under a non-reducing condition (Figure 3C). To confirm that the ERO1-α-PDI pathway oxidized PD-L1, we examined the redox status of ERO1-α, PDI, and PD-L1 by Western blotting. We found that the ERO1-α-PDI complex in treated cells was decreased compared with that in control cells under a non-reducing condition. Moreover, EN460 treatment resulted in conversion of the oxidized form of ERO1-α to the reduced form, indicating loss of function for re-oxidation of PDI [36]. These results again suggested that the expression of PD-L1 is dependent on the oxidative folding activity of ERO1-α.

### Knockdown of ERO1-α in MDA-MB-231 cells decreases the rate of apoptosis of Jurkat leukemia T cells

**Figure 4:**
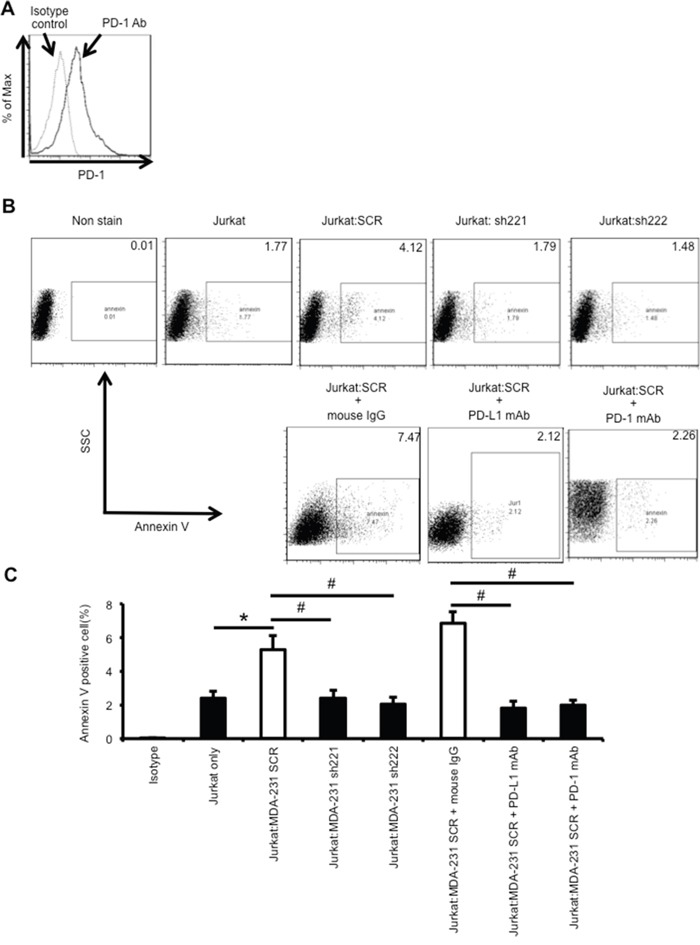
Knockdown of ERO1-α decreased the rate of apoptosis of Jurkat T cells by decreasing PD-L1 expression on tumor cells **A**. Flow cytometric analysis of PD-1 expression on Jurkat cells (solid line). Jurkat cells incubated with an APC-labeled isotype control served as background controls (dotted line). **B, C**. MDA-MB-231 SCR cells and KD (sh221, sh222) cells were cocultured with Jurkat leukemia T cells. Cocultures (tumor cell to Jurkat cell ratio =10:1) were incubated with mouse IgG (2 or 5 μg/ml; IBL), anti-human PD-L1 antibody (5 μg/ml; BD) or anti-PD-1 antibody (2 μg/ml; BioLegend) for 24 hours. To block the PD-1/PD-L1 pathway, SCR cells were preincubated with anti-human PD-L1 antibody (5 μg/ml) for 30 min or Jurkat cells were preincubated with anti-PD-1 antibody (2 μg/ml) for 30 min. Extent of apoptosis in Jurkat cells was determined by flow cytometry using FITC-Annexin V. SSC: side scatter. The experiment was repeated three times with essentially the same results. *p<0.05, unpaired Student's t-test. ^#^p<0.05, Dunnett's test. Data are shown from single experiments representative of three experiments performed.

Jurkat leukemia T cells expressed PD-1 (Figure 4A), which is known as a receptor for PD-L1. We observed that coculture of Jurkat cells with PD-L1-positive MDA-MB-231 SCR cells resulted in apoptotic cell death (Figure 4B, 4C). To examine the effect of ERO1-α on the function of PD-L1, SCR and KD cells (sh221, sh222) were cocultured with Jurkat leukemia T cells. We found that the rate of apoptosis in Jurkat T cells cocultured with SCR cells was higher than that in Jurkat cells only (Figure 4B, 4C). We also found that the rate of apoptosis in Jurkat T cells cocultured with KD cells was lower than that in Jurkat T cells cocultured with SCR cells. On the other hand, the rate of apoptosis in Jurkat T cells cocultured with SCR cells was decreased by treatment with an anti-PD-L1 or anti-PD-1 mAb. These results suggested that the expression of ERO1-α in tumor cells upregulated PD-L1 expression, resulting in the induction of apoptosis of tumor-infiltrating T cells.

### Expression of ERO1-α in a tumor is correlated with expression of PD-L1 in clinical cases

**Figure 5:**
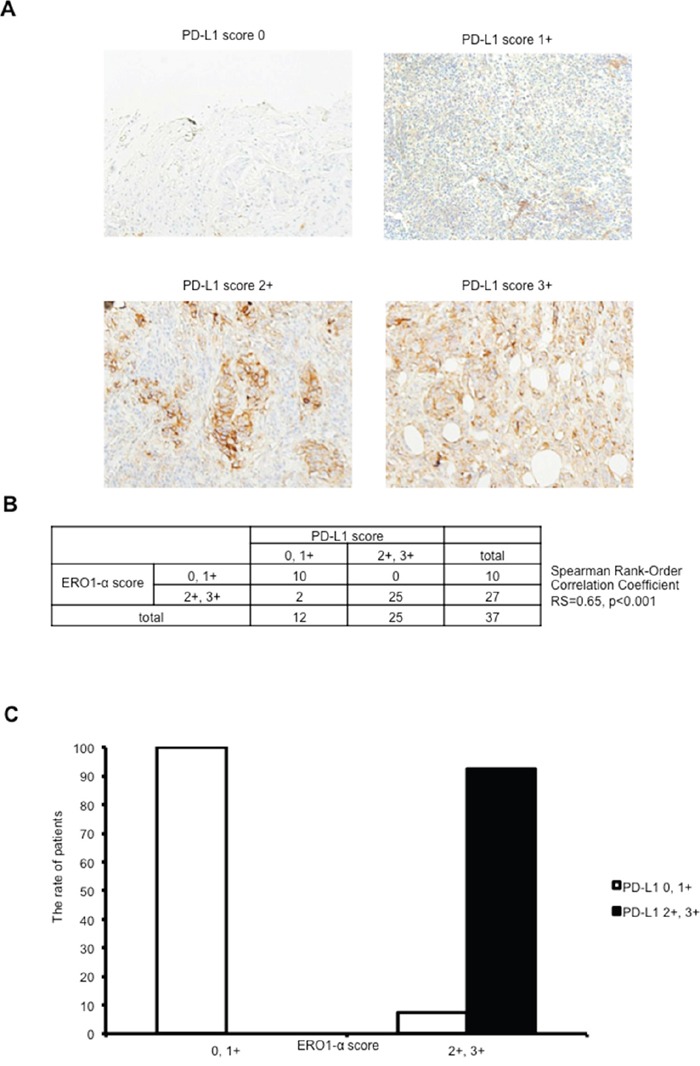
Expression of ERO1-α in triple negative breast cancer was correlated with the expression of PD-L1 in clinical cases **A**. Tumors were classified into 4 groups by stainability for PD-L1. **B**. Tumors were analyzed for correlation between the expression of ERO1-α and expression of PD-L1 by Spearman's rank-order correlation coefficient. **C**. The ERO1-α score 0, 1+ group showed significantly reduced expression of PD-L1 compared with that in the ERO1-α score 2+, 3+ group.

To examine the correlation between expression of ERO1-α and expression of PD-L1 in clinical cases, TNBC tissues were stained with an anti-ERO1-α mAb and anti-PD-L1 mAb, and they were classified into four groups based on the expression status (Figure 5A). It was found the expression level of ERO1-α was significantly correlated with the expression of PD-L1 (RS=0.65, P < 0.001) (Figure 5B). The ERO1-α score 0, 1+ group showed significantly reduced expression of PD-L1 compared with that in the ERO1-α score 2+, 3+ group (Figure 5C). These results indicated that enhanced expression of ERO1-α in TNBC plays a critical role in the expression of PD-L1.

## DISCUSSION

During tumor progression, tumors must develop strategies to evade antitumor immunity. Moreover, hypoxia, a hallmark of the tumor microenvironment, contributes to the acquisition of more aggressive properties in cancer cells such as drug resistance and metastatic ability. Among oxidoreductases, ERO1-α is unique, because it is upregulated within tumor cells [22–25] and under the condition of hypoxia [37, 38]. Oxidative protein folding such as intramolecular disulfide bond formation is the most common post-transcriptional modification [20]. Proper disulfide bond formation is required for correct conformation and function of proteins [21]. Most of the proteins that are secreted from cells or expressed on the cell surface have intramolecular disulfide bonds. Therefore, oxidative protein folding is critical for the cells to function and survive. ERO1-α plays a central role in oxidative protein folding in collaboration with PDI [21, 39]. We previously showed that ERO1-α is overexpressed in various types of cancer cell lines and cancer tissues including breast cancer tissues compared with levels in normal cells and normal tissues [22–25]. Moreover, we reported that overexpression of ERO1-α within the tumor was a poor prognostic factor in patients with breast cancer [24]. In this study, we found by using triple negative breast cancer MDA-MB-231 cells and MDA-MB-468 cells that overexpression of ERO1-α protein resulted in augmented expression of PD-L1. It is known that disulfide bond formation by ERO1-α is accompanied by the production of hydrogen peroxide (H_2_O_2_), a potential source of ROS [28]. ROS increase the HIF-1α protein level via inhibition of enzymatic activity of a family of prolyl hydroxylases [26, 27]. In fact, we showed that overexpression of ERO1-α increased levels of ROS (Supplementary Figure 1D, 1E), resulting in increased HIF-1α protein levels and both mRNA and protein levels of PD-L1. These results coincide with results of recent studies showing that HIF-1α regulates PD-L1 expression [8, 9]. Moreover, since PD-L1 protein has an intramolecular disulfide bond [29], we examined the redox status of PD-L1. As expected, the expression of ERO1-α facilitated oxidative folding of PD-L1, resulting in an increase in the oxidized form of PD-L1. In contrast, knockdown or functional inhibition of ERO1-α resulted in decreased expression of PD-L1 only at post-transcriptional levels. It has been shown that the PD-L1 mRNA expression level is regulated by NF-κ [30], IRF-1 [31], STATs [32, 33], MEK/ERK [32, 34] and AP-1 [30]. Additionally, HIF-1α enhances PD-L1 mRNA expression under certain conditions including hypoxia [8, 9]. Our results suggest that low or moderate levels of HIF-1α observed in WT cells, SCR cells, and KD cells had no effect on PD-L1 expression, indicating that PD-L1 expression is regulated by factors other than HIF-1α in these cells. However, high levels of HIF-1α detected in OE cells play a very important role in PD-L1 mRNA expression. The precise mechanism by which PD-L1 expression is regulated must be clarified in future studies. Thus, ERO1-α affected PD-L1 expression at both mRNA levels and posttranscriptional levels (Supplementary Figure 5).

Recently, it has been demonstrated that ERO1-α-derived H_2_O_2_ is cleared by the ER-resident peroxidases glutathione peroxidase (GPx) 7/8 and peroxiredoxin (Prx) IV [40, 41]. However, we found high levels of ROS within OE cells. These results suggest that high levels of ROS produced by the action of ERO1-α in OE cells might be beyond the ROS-detoxifying ability of GPx7/8 and Prx IV, resulting in the accumulation of ROS and HIF-1α protein as well as increased expression of PD-L1 mRNA.

In this study using human TNBC cell lines, we showed that ERO1-α plays an important role in the expression of PD-L1 via facilitation of oxidative protein folding and in the upregulation of PD-L1 mRNA via transcriptional activity of HIF-1α. Importantly, downregulation of PD-L1 expression by knockdown of ERO1-α resulted in decreased PD-L1-induced apoptosis of T-cells. Therefore, it should be clarified whether targeting ERO1-α in tumor cells results in augmentation of T-cell-mediated tumor cell killing *in vitro* as well as in an immunocompetent mouse model of breast cancer.

## MATERIALS AND METHODS

### Cells and agents

Jurkat leukemia T cells and human breast cancer lines MDA-MB-231 and MDA-MB-468 were purchased from ATCC (Manassas, VA, USA). The cell lines were authenticated by the ATCC using short tandem repeat profiling and passaged in our laboratory for fewer than 6 months after receipt. Jurkat leukemia T cells were cultured in RPMI-1640 (Sigma-Aldrich, St. Louis, MO, USA) supplemented with 10% FCS at 37°C in 5% CO_2_. MDA-MB-231 and MDA-MB-468 cells were cultured in Dulbecco's modified Eagle's medium (Sigma-Aldrich) supplemented with 10% FCS at 37°C in 5% CO_2_. Short hairpin RNA for human ERO1-α (TR313168) was purchased from OriGene (Rockville, MD, USA) and transfected to MDA-MB-231 cells using Lipofectamine 2000 (Life Technologies). To establish ERO1-α-overexpressing cells, MDA-MB-231 cells were transfected with pIRES puro3 myc2/ERO1 or an empty vector as a control using Lipofectamine 2000 (Life Technologies) per the manufacturer's instructions. Cells were stably propagated under puromycin selection (2 μg/ml). siRNA to ERO1-α and control siRNA were purchased from Origene and transfected to MDA-MB-468 cells. Four days after transfection, cells were harvested and used in flowcytometric assay and gene expression assay. SiRNA to HIF-1α and control siRNA were purchased from Origene and transfected to MDA-MB-231 cells.

### Inhibition of ERO1-α function by using the ERO1-α inhibitor EN460

The ERO1-α inhibitor EN460 (328501) was purchased from Millipore (Billerica, MA, USA). MDA-MB-231 WT cells were plated at 5 × 10^5^ cells / well in 6-well plates and were incubated with 12.5 μM EN460 for 24 h.

### Real-time PCR analysis

Total RNA was isolated from cultured cells and normal breast tissues using Trizol reagent (Life Technologies) and RNeasy Mini kits (QIAGEN, Valencia, CA) according to the instructions of the manufacturers. The cDNA mixture was synthesized from 1 μg total RNA by reverse transcription using Superscript III and oligo (dT) primer (Life Technologies) according to the manufacturer's protocol. PCR amplification was performed in 20 μl of PCR mixture containing 1 μl of cDNA mixture, 0.1 μl of Taq DNA polymerase (QIAGEN) and 6 pmol of primers. Real-time relative polymerase chain reaction (real-time PCR) was performed to determine the expression levels of ERO1-α, PD-L1 and β-actin. Expression values for each sample were normalized to β-actin, and fold levels of the indicated genes represent the mean (±SEM) of replicate reactions. Primer sequences were as follows: β-actin (ACTB), Hs0160665_g1; ERO1-α (ERO1L), Hs00205880_m1; and PD-L1, Hs01125301_m1 (Life Technologies). PCR cycles were performed on the StepOne Real-Time PCR System (Life Technologies) with the following cycle conditions: 2 min at 50 °C, 10 min at 95°C, 45 cycles of 15 s at 95°C and 1 min at 60°C. The delta-delta Ct method was used for data analysis.

### Western blot analysis

Cultured cells were washed in ice-cold PBS, lysed by incubation on ice in a lysis buffer (50 mmol /L Tris-HCl, 150 mmol /L NaCl, 5 mmol/L EDTA, 1% NP40), and cleared by centrifugation at 21880 g for 30 min at 4°C. For blockade of free thiols, cells were pretreated for 5 min with 20 mM N-ethylmaleimide (NEM) (Thermo Fisher Scientific) or 10 mM methyl methanethiosulfonate (MMTS) (23011; Pierce, Rockford, IL, USA) in PBS. Post-nuclear supernatants were divided and heated for 5 min at 95°C in a non-reducing or reducing SDS sample buffer, resolved by SDS-PAGE, and electrophoretically transferred to PVDF membranes (Immobilon-P; Millipore, Billerica, MA, USA). The membranes were incubated with blocking buffer (5% non-fat dried milk in PBS) for 30 min at room temperature and then incubated overnight with anti-ERO1-α mAb (H00030001-M01; Abnova, Taipei, Taiwan), anti-PD-L1 mAb (ab174838; Abcam), anti-PDI pAb (ADI-SPI-890; Enzo Life Sciences, Farmingdale, NY, USA) or mouse anti-β-actin mAb (AC-15; Sigma-Aldrich, St. Louis, MO, USA). After washing three times with wash buffer (0.1% Tween-20 in TBS), the membranes were reacted with peroxidase-labeled goat anti-mouse IgG (074-1806; KPL, Gaithersburg, MD, USA) antibody or peroxidase-labeled goat anti-rabbit IgG antibody (074-1516; KPL) for 3 h. Finally, the signal was visualized using an ECL detection system (GE Healthcare UK Ltd., Amersham Place, Little Chalfont, Buckinghamshire, England) according to the manufacturer's protocol.

### Analysis of the level of ROS

Cells were collected by trypsinization, washed with DMEM twice, resuspended in FCS-free DMEM with 10 μM of 5-(and-6)-chloromethyl-2′,7′-dichlorofluorescine diacetate acetyl ester (CM-H_2_DCFDA) (C6827; Life Technologies), and incubated at 37 °C for 30 min in the dark. Then the fluorescence intensity of the cells was determined by using a FACSCalibur flow cytometer (BD, San Jose, CA) and FlowJo (Tree Star Inc., Oregon, USA). For the detection of intracellular ROS levels, cells were plated at 1 × 10^5^ cells / well in 35-mm glass bottom dish and incubated for 24 h. Medium was replaced by FCS-free DMEM with 10 μM of CM-H_2_DCFDA (Life Technologies), and incubated at 37 °C for 2 h in the dark. Then fluorescence intensity was determined by using fluorescent microscopy (Olympus, Japan).

### Immunohistochemistry

Tissue samples were obtained from 37 patients diagnosed with triple negative breast cancer in 2005 at Sapporo Medical University Hospital in Sapporo, Japan. The expression of PD-L1 and ERO1-α at the protein levels were evaluated by immunohistochemical analysis. Tissue was fixed in neutral 10% buffered formaldehyde, embedded in paraffin, and cut into 5-μm-thick slices for ERO1-α staining and PD-L1 staining. Reactivity of the anti-ERO1-α mAb was determined by perinuclear staining within tumor cells, indicating endoplasmic reticulum localization. The expression status of ERO1-α was graded by our original classification: score 0 (positive cells: 0%), score 1 (positive cells: < 10%), score 2 (10% ≦ positive cells ≦ 30%), score 3 (positive cells: > 30%). The expression status of PD-L1 was graded by our original classification: score 0 (positive cells: 0%), score 1 (positive cells: < 30%), score 2 (30% ≦ positive cells ≦ 80%), score 3 (positive cells: > 80%).

### Flow cytometric analysis

Cells were collected by trypsinization and washed with ice-cold PBS. The cells were then stained with PE-labeled anti-PD-L1 mAb (29E. 2A3; BioLegend, San Diego, CA), APC-labeled anti-PD-1 mAb (EH12.2H7; BioLegend) or isotype control and analyzed by flow cytometry. Labeled cells were analyzed by a FACSCalibur flow cytometer (BD, San Jose, CA) and FlowJo (Tree Star Inc., Oregon, USA).

### Coculture study and assessment of apoptosis

To examine the effect of tumor cells on lymphocyte apoptosis, 5×10^6^ MDA-MB-231 SCR cells and KD cells were cocultured with 5×10^5^ Jurkat leukemia T cells in the presence of mouse IgG (2 or 5 μg/ml; 17314; IBL Co., Ltd., Gunma, Japan), anti-human PD-L1 antibody (5 μg/ml; MIH1; BD) or anti-PD-1 antibody (2 μg/ml; EH12.2H7; BioLegend) in a 6-well plate for 24 hours. To block the PD-1/PD-L1 pathway, SCR cells were preincubated with anti-human PD-L1 antibody (5 μg/ml; BD) for 30 min or Jurkat cells were preincubated with anti-PD-1 antibody (2 μg/ml; BioLegend) for 30 min. Extent of apoptosis in Jurkat cells was determined by flow cytometry using FITC-Annexin V (11858777001; Roche) according to the manufacturer's instructions.

### Statistical analysis

Student's *t*-test or Welch's *t*-test was used for analysis of two unpaired samples. Dunnett's test was used for analysis of multiple samples. All analyses were carried out with STATMATE version 3.19 (ATMS Co., Ltd., Tokyo, Japan). A P-value of less than 0.05 was regarded as statistically significant. All statistical tests were two-sided.

## SUPPLEMENTARY MATERIALS FIGURES AND TABLES



## Abbreviations

ER: endoplasmic reticulum
ERO1-α: endoplasmic reticulum oxidoreductase 1-α
H_2_O_2_: hydrogen peroxide
HER2: HER2/neu
HIF-1α: hypoxia-inducible factor-1α
KD: ERO1-α knockdown
MMTS: methyl methanethiosulfonate
OE: ERO1-α-overexpression
PD-1: programmed cell death-1
PDI: protein disulfide isomerase
PD-L1: programmed death-ligand 1
PHD: prolyl hydroxylase
ROS: reactive oxygen species
SCR: scramble
TNBC: triple negative breast cancer
NEM: N-ethylmaleimide
