# Supplementary material for: Cancer-associated oxidoreductase ERO1-α promotes immune escape through up-regulation of PD-L1 in human breast cancer
Source: Oncotarget. 2017 Feb 1;8(15):24706–18. doi: 10.18632/oncotarget.14960 (PMC5421881; doi:10.18632/oncotarget.14960)
Supplement: Supplementary file 1 [file oncotarget-08-24706-s001.pdf]

Cancer-associated oxidoreductase ERO1-α promotes immune escape through up-regulation of PD-L1 in human breast cancer

SUPPLEMENTARY FIGURES

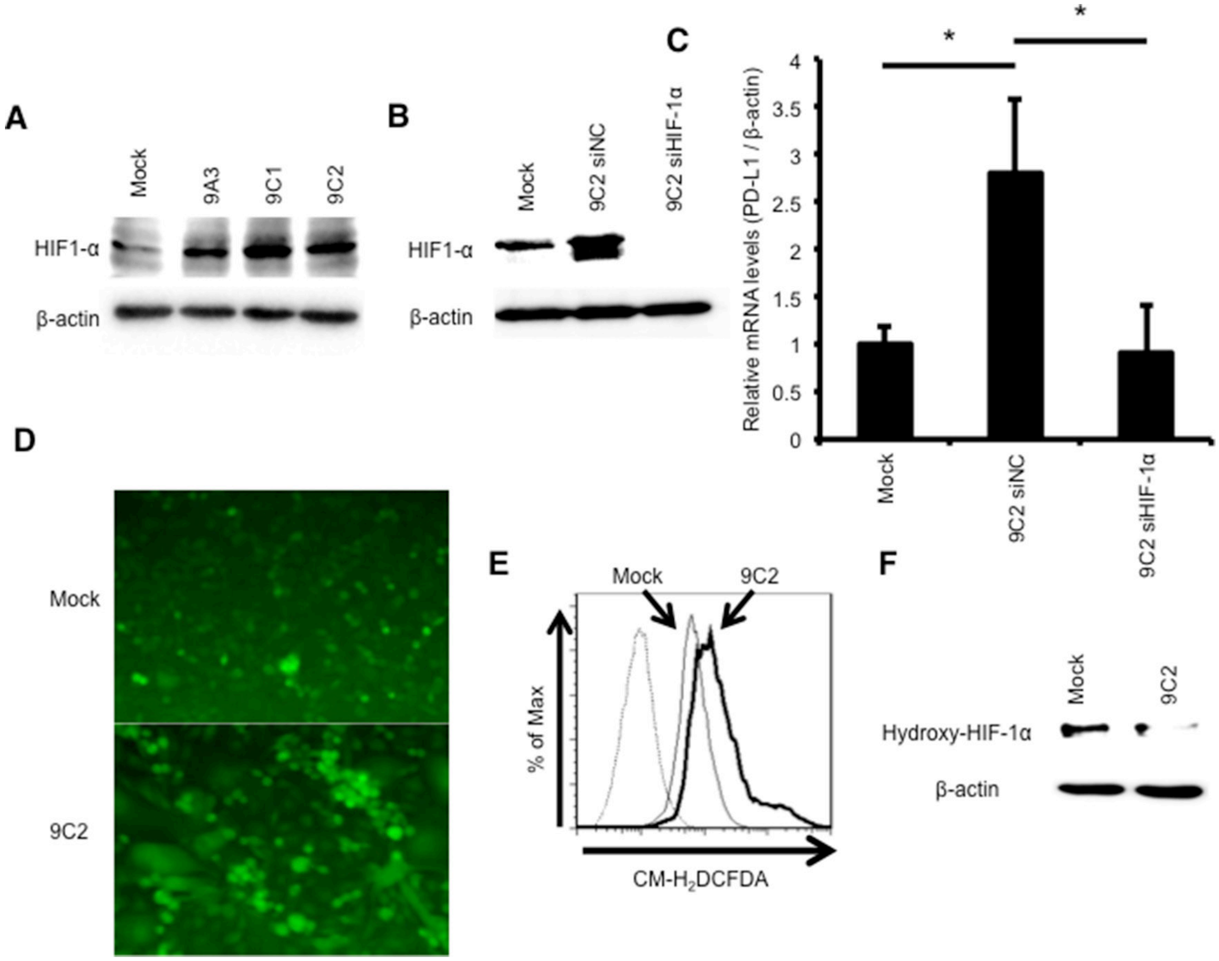

Supplementary Figure 1: Overexpression of ERO1-α upregulates PD-L1 expression via HIF-1α, which is accumulated by ROS. **A.** Western blot analysis of HIF-1α in MDA-MB-231 mock and ERO1-α-overexpressed (OE: 9A3, 9C1 and 9C2) cells. **B.** Western blot analysis of HIF-1α in mock, 9C2 siNC and 9C2 siHIF-1α cells. **C.** PD-L1 mRNA levels in mock, 9C2 siNC and 9C2 siHIF-1α cells determined by real-time PCR. **D.** Intracellular ROS levels of mock and 9C2 cells detected using CM-H_2DCFDA. **E.** Levels of ROS detected by CM-H_2DCFDA staining within 9C2 cells (bold line) was compared with that in mock cells (thin line) using flow cytometric analysis. These cells incubated with DMSO served as background controls (dotted line and dashed line). **F.** The levels of hydroxyl-HIF-1α in mock and 9C2 cells were compared by Western blotting. * $p<0.05$, unpaired Student's t-test.

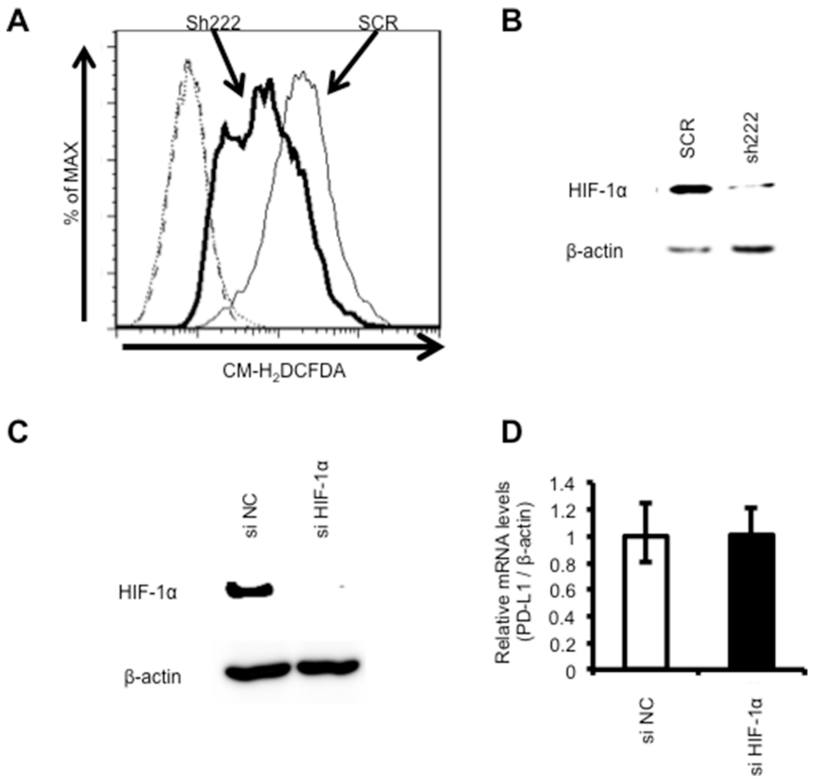

Supplementary Figure 2: A. Levels of ROS detected by CM-H_2DCFDA staining within MDA-MB-231 ERO1-α knockdown (sh222) cells (bold line) was compared with that in MDA-MB-231 scrambled shRNA-transfected (SCR) cells (thin line) using flow cytometric analysis. These cells incubated with DMSO served as background controls (dotted line and dashed line). **B.** Western blot analysis of HIF-1α in SCR and sh222 cells. **C.** Western blot analysis of HIF-1α in MDA-MB-231 scrambled siRNA-transfected (siNC) and HIF-1α knockdown (siHIF-1α) cells. **D.** PD-L1 mRNA levels in siNC and siHIF-1α cells determined by real-time PCR.

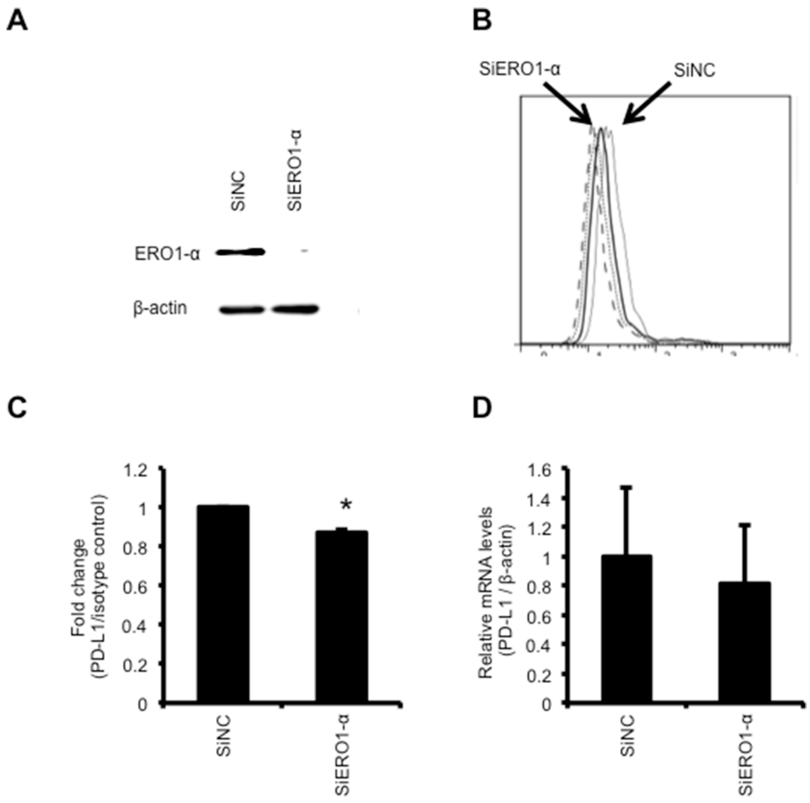

Supplementary Figure 3: Knockdown of ERO1-α downregulates PD-L1 expression via decreased oxidative protein folding. A. Western blot analysis of ERO1-α in MDA-MB-468 scrambled siRNA-transfected (siNC) and ERO1-α knockdown (siERO1-α) cells. **B.** Flow cytometric analysis of PD-L1 expression on siNC cells (thin line) and siERO1-α cells (bold line). These cells incubated with a PE-labeled isotype control served as background controls (dotted line and dashed line). **C.** The mean fluorescence intensity (MFI) value obtained in siNC cells was set to 1, and differences in MFI caused by siERO1-α cells were plotted. **D.** PD-L1 mRNA levels in siNC and siERO1-α cells determined by real-time PCR. * $p<0.001$, unpaired Student's t-test.

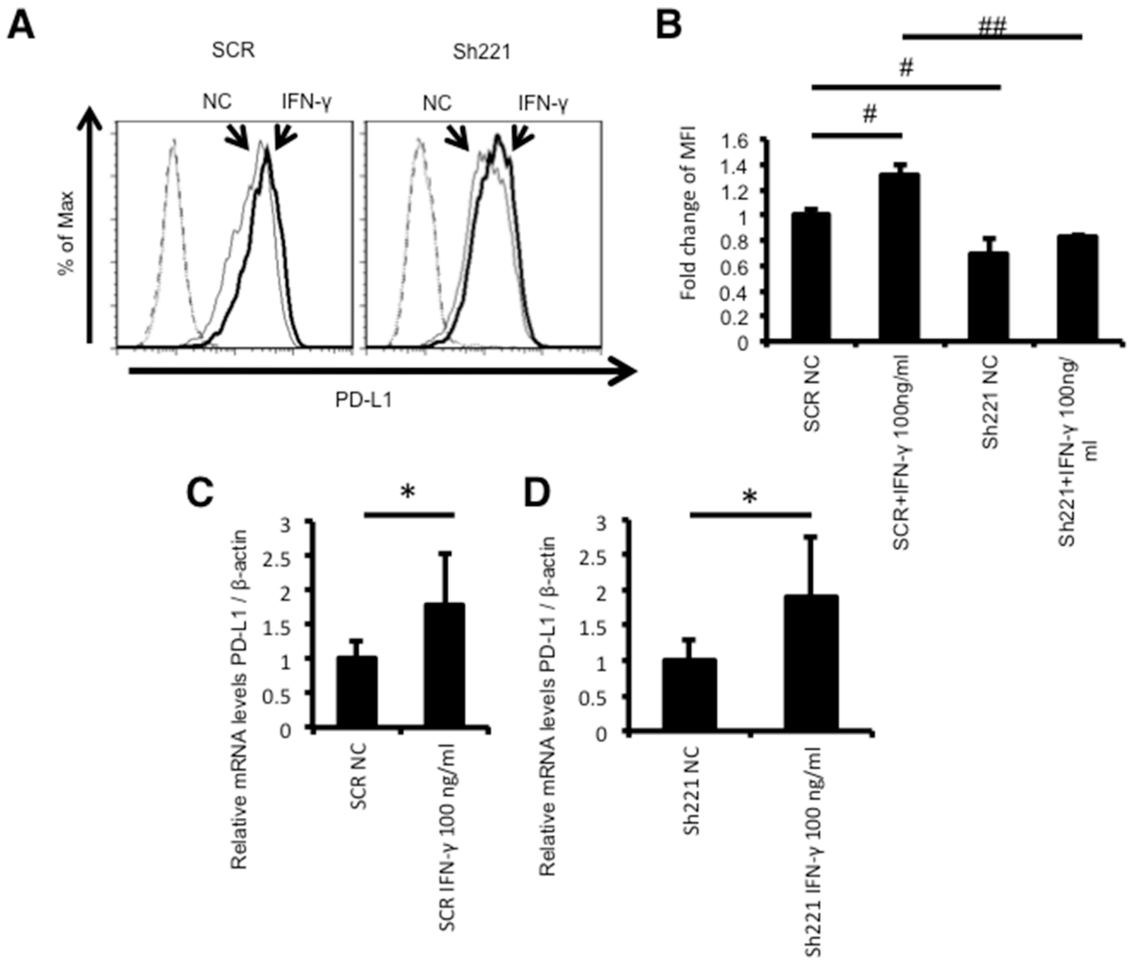

Supplementary Figure 4: Role of ERO1-α in IFN-γ-induced upregulation of PD-L1 expression. A. Flow cytometric analysis of PD-L1 expression on SCR cells and SCR cells treated with IFN-γ (left column) and on sh221 KD cells and sh221 KD cells treated with IFN-γ (right column). These cells incubated with a PE-labeled isotype control served as background controls (dotted line and dashed line). **B.** The mean fluorescence intensity (MFI) value obtained in SCR NC cells was set to 1, and differences in MFI caused by SCR cells treated with IFN-γ, sh221 NC cells, and sh221 cells treated with IFN-γ were plotted. **C, D.** PD-L1 mRNA levels in SCR cells and SCR cells treated with IFN-γ and in sh221 KD cells and sh221 cells treated with IFN-γ determined by real-time PCR. $^{\#}p<0.01$, Dunnett's test, $^{\#\#}p<0.01$, Welch's test. $^{*}p<0.05$, unpaired Student's *t*-test.

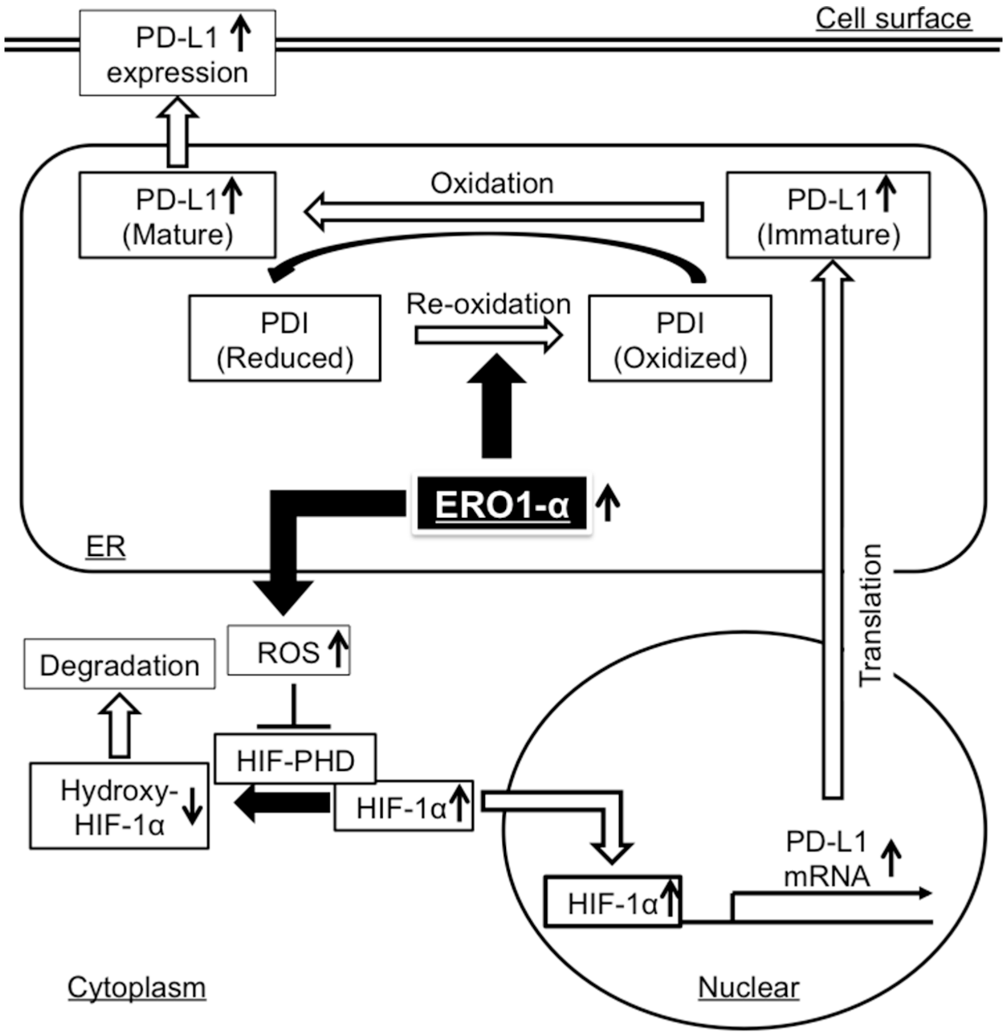

Supplementary Figure 5: Schematic model of influence of ERO1-α for PD-L1 expression. (1) ERO1-α produces ROS when ERO1-α reoxidizes reduced PDI. (2) ROS regulate the degradation of HIF-1α via inhibiting the function of HIF-PHD. (3) Accumulated HIF-1α increases PD-L1 mRNA expression levels and protein levels. (4) Immature PD-L1 is matured by oxidative protein folding on the ERO1-α-PDI pathway.
